# Old World camels in a modern world – a balancing act between conservation and genetic improvement

**DOI:** 10.1111/age.12858

**Published:** 2019-09-18

**Authors:** P. A. Burger, E. Ciani, B. Faye

**Affiliations:** ^1^ Research Institute of Wildlife Ecology Vetmeduni Vienna Vienna 1160 Austria; ^2^ Dipartimento di Bioscienze, Biotecnologie e Biofarmaceutica Università degli Studi di Bari ‘Aldo Moro’ Via Orabona, 4 70125 Bari Italy; ^3^ CIRAD‐ES UMR SELMET TAC/112A Campus international de Baillarguet 34398 Montpellier cedex France

**Keywords:** Bactrian camel, domestication, dromedary, evolutionary history

## Abstract

Old World camels have served humans in cross‐continental caravans, transporting people and goods, connecting different cultures and providing milk, meat, wool and draught since their domestication around 3000–6000 years ago. In a world of modern transport and fast connectivity, these beasts of burden seem to be out‐dated. However, a growing demand for sustainable milk and meat production, especially in countries affected by climate change and increasing desertification, brings dromedaries (*Camelus dromedarius*) and Bactrian camels (*Camelus bactrianus*) back onstage and into the focus of animal breeders and scientists. In this review on the molecular genetics of these economically important species we give an overview about the evolutionary history, domestication and dispersal of Old World camels, whereas highlighting the need for conservation of wild two‐humped camels (*Camelus ferus*) as an evolutionarily unique and highly endangered species. We provide cutting‐edge information on the current molecular resources and on‐going sequencing projects. We cannot emphasise enough the importance of balancing the need for improving camel production traits with maintaining the genetic diversity in two domestic species with specific physiological adaptation to a desert environment.

## Introduction

Increasing desertification owing to global climate change and the growing demand for sustainable meat and milk production challenge the field of animal breeding and livestock science. The two domesticated Old World camel species, one‐humped dromedaries (*Camelus dromedarius*) and two‐humped Bactrian camels (*Camelus bactrianus*) seem to be a perfect answer to these challenges as they are resilient to harsh climatic conditions and highly efficient in their production (Faye & Konuspayeva 2012).

In this review, we aim to provide a comprehensive summary of the evolutionary history and domestication of Old World camels as well as their global dispersal. We will discuss the historic and ongoing hybridisation between Old World camels, which serves as improvement of production traits (milk and wool) in the domesticated dromedary and Bactrian camels, but threatens the genetic integrity of the last existing, highly endangered, wild two‐humped camels (*Camelus ferus*). Finally, the purpose and main types including interesting traits for performance and adaptation, will be discussed, as well as the currently available molecular resources to investigate these traits. We emphasise that it is important to keep a balance between conserving the genetic integrity, diversity and traditional management of the species, while responding to the constantly growing needs for intensification of breeding and selection using modern genomic tools.

## Evolutionary history and domestication of Old World camels

### Evolutionary history of Old World camels

**Figure 1:**
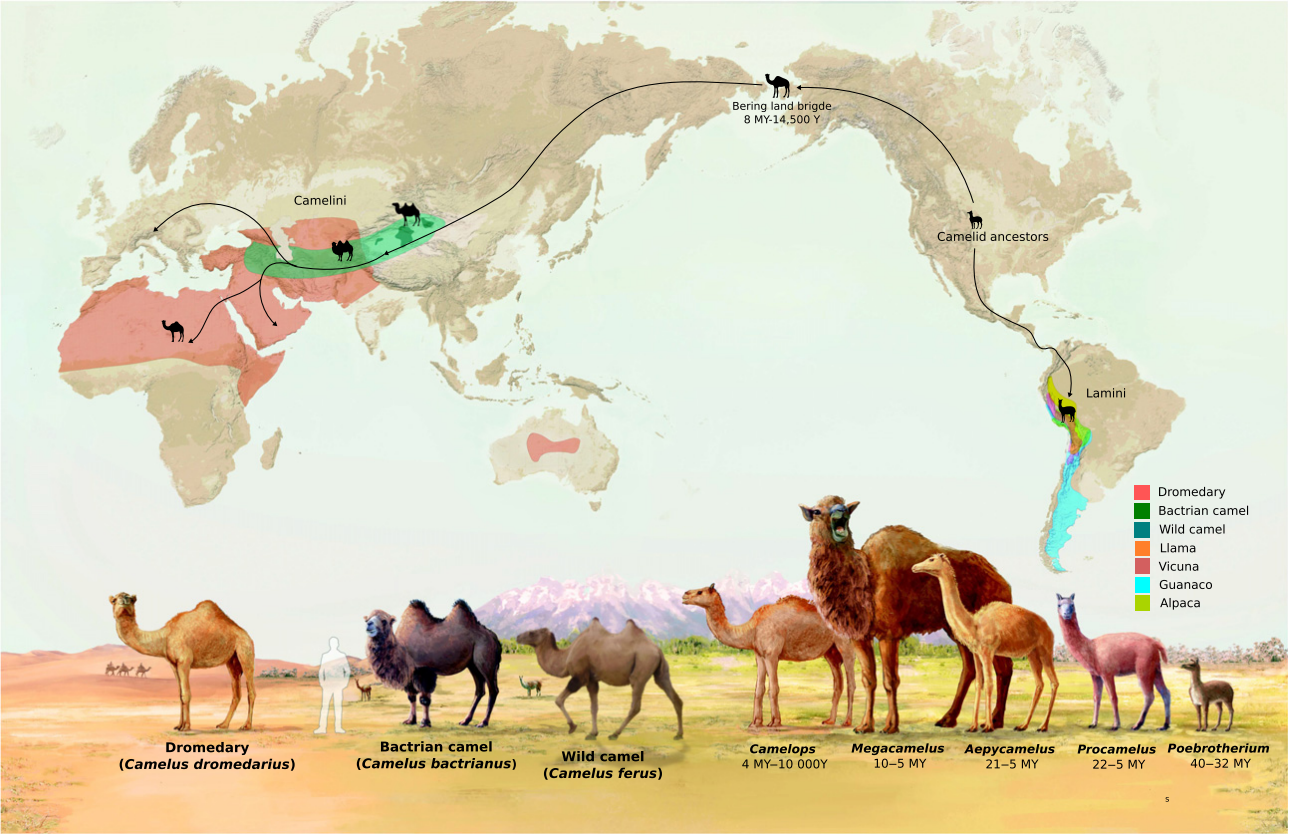
Migration map of the historical camelid family. The current distribution of dromedaries and Bactrian camels is presented in red and green colours. The last refugia of the wild two‐humped camels in China and Mongolia are shown as dark‐green patches. The map was adapted from Mesa Schumacher/AramcoWorld (https://www.aramcoworld.com/en-US/Articles/November-2018/The-Magnificent-Migration). Reprint permits were granted by AramcoWorld on March 6, 2019.

Modern camels belong to the order of Artiodactyla (even‐toed ungulates), suborder Tylopoda, and the family of Camelidae consisting of the tribes Camelini (Old World camels) and Lamini (New World camels), which diverged 16.3 (9.4–25.3) million years ago (Mya; Wu *et al*. 2014). Similar to other large mammals, the earliest‐known ancestors of the camelid family, Protylopus, originated in the North American savannah during the Eocene (~45 Ma), with a size smaller than a goat. At least 20 genera of camelids, e.g. Megacamelus and Procamelus, developed and disappeared again over the following million years (Honey *et al*. 1998; Rybczynski *et al*. 2013), until the ancestors of Old World camels reached Eurasia via the Bering land bridge around 6.5–7.5 Mya. Fossils of Paracamelus and other giant camels have been recorded in Asia (Kozhamkulova 1986; Flynn 1997), Europe (e.g. Spain; Pickford *et al*. 1995), Northern Africa (*Camelus thomasi*; Peters 1998) and the Arabian Peninsula (e.g. Syria; Martini *et al*. 2015). The progenitors of the New World camels entered South America around 3 Mya (Prothero & Schoch 2002; Rybczynski *et al*. 2013). Figure 1 presents an early migration map of camels.

Within Camelini, three species are recognised today based on the International Commission on Zoological Nomenclature (2003) (Gentry *et al*. 2004) and genomic evidence: the domesticated *C. dromedarius* and *C. bactrianus*, and the only remaining wild species, the two‐humped *C. ferus*. Whereas one‐ and two‐humped camels diverged 4.4 (1.9–7.2) Mya (Wu *et al*. 2014), the split between the ancestors of wild and domestic Bactrian camels is more recent and was estimated at 1.1 (0.6–1.8) Mya (Ji *et al*. 2009; Mohandesan *et al*. 2017).

Wild two‐humped camels, discovered by Nikolaj Przewalski in 1878, might have been distributed throughout Central Asia but reconstruction of their original distribution is difficult owing to scarcity of bone remains from archaeological sites, rock art and historical writings (Peters & von den Driesch 1997). Nowadays their range has become severely reduced to only four locations worldwide: three in China (Taklamakan desert, Gashun Gobi desert and Arjin Mountains in the Lop Nur Lake region) and one in Mongolia (Great Gobi Strictly Protected Area ‘A’). These are now the last refuges for wild camels, which are listed as Critically Endangered (Hare 2008), as estimates for the numbers of remaining animals range from 1000 to 1600 (Lei *et al*. 2012; Yadamsuren *et al*. 2012). The genetic status of *C. ferus* has been heavily debated, as morphological similarities with its domestic counterpart led to the assumption that wild camels were merely the descendants of domestic animals that had returned to the wild (Peters & von den Driesch 1997). However, the International Commission on Zoological Nomenclature (2003) fixed the first available specific name based on a wild population ‘*C. ferus*’ to the wild camel (Gentry *et al*. 2004), and genetic studies (Ji *et al*. 2009; Silbermayr *et al*. 2010; Jirimutu *et al*. 2012; Mohandesan *et al*. 2017; Yi *et al*. 2017) confirmed that the wild two‐humped camel is an original wild form and separate species *C. ferus*.

### Domestication of Bactrian camels

Based on archaeological and pictorial evidence, the period of Bactrian camel domestication has been estimated as beginning in the late fourth and early third millennium before common era (BCE; Bulliet 1975; Benecke 1994). The regions of domestication, however, are still a matter of debate, with two hypotheses presently being discussed: H_I_, domestication took place in northeastern Iran and the adjacent Kopet Dagh foothills in southwestern Turkmenistan, part of the historical region ‘Bactria’, which was eponymous for domestic two‐humped camels (Beneke 1994); and H_II_, a centre of domestication was further to the east where people were familiar with wild camels over an extended period of time, e.g. in Kazakhstan or northwestern Mongolia. The lack of wild camel remains in the Neolithic strata of the Kopet Dagh foothills led to the assumption that fully domesticated two‐humped camels were acquired from eastern Asia (Peters & von den Driesch 1997). Both hypotheses still need to be tested using ancient DNA analyses of wild and early‐domestic two‐humped camel samples. A preliminary palaeogenetic analysis of 12 Bactrian camel bones from Late Bronze and Early Iron Age sites in Uzbekistan and Siberia showed the same mitochondrial haplotypes as described in modern domestic Bactrian camels, suggesting a single domestication process (Trinks *et al*. 2012). A significantly higher genetic diversity detected in the genomes of Iranian camels could hint of an ancient origin of domestic Bactrian camels from this region (Jirimutu & Ming 2018), supporting hypothesis H_I_. However, post‐domesticated cross‐species hybridisation with dromedaries would have left similar signals in the genomes and thus cannot be ruled out. Movements of domestic camels or multiple origins of the founder populations also could have led to the observed higher diversity in Iranian camels.

### Domestication of dromedaries

**Figure 2:**
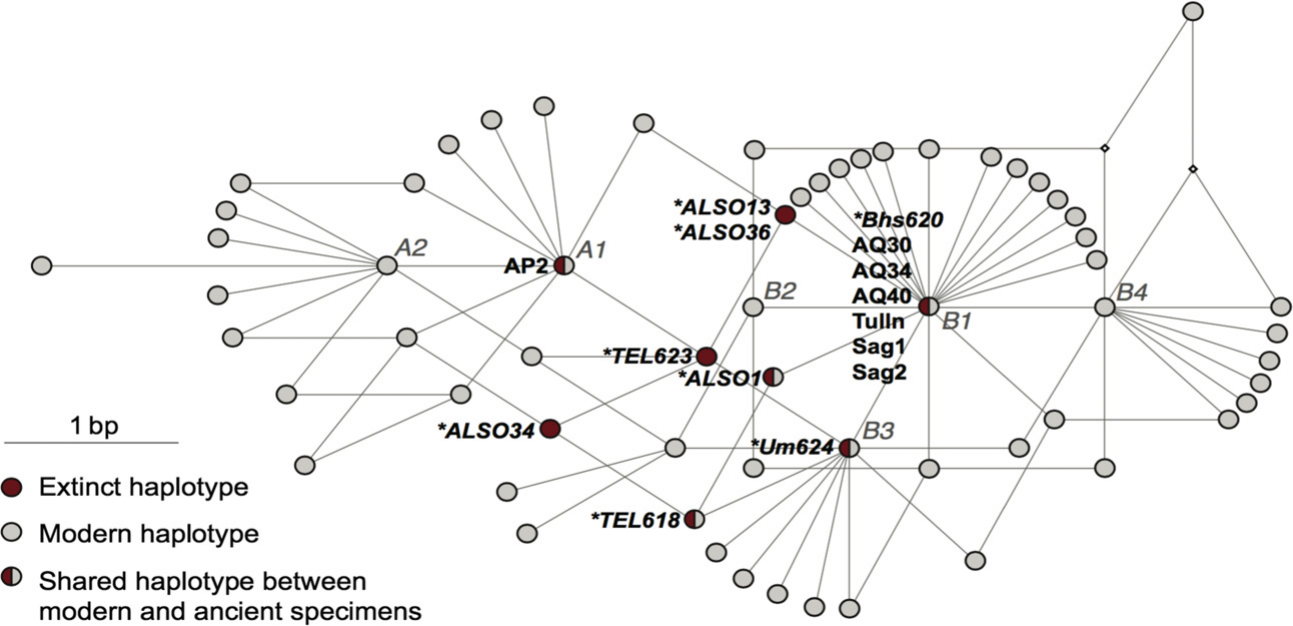
Mitochondrial DNA neighbour joining network of ancient (red) and modern (grey) dromedary samples. Wild extinct dromedary samples are marked with an asterisk. The geographical origin of the archaeological specimen is shown in Fig. 3. Reprinted from Supplementary Material of Almathen *et al*. (2016).

Based on osteological and pictorial evidence as well as cultural context, the domestication of dromedaries probably happened in the late second millennium (1100–1800) BCE (Uerpmann & Uerpmann 2002; von den Driesch & Obermaier 2007; Iamoni 2009 and Grigson 2012, 2014; Uerpmann & Uerpman 2012; Magee 2015). Mitochondrial, nuclear and ancient DNA analyses of a global dataset of modern individuals and up to 7000‐year‐old wild dromedary samples revealed shared ancestry between wild dromedaries from the southeast coast of the Arabian Peninsula and modern animals (Fig. 2). A minimum of six wild maternal lineages were captured during the process of domestication with the most frequent mitochondrial haplotype still present in approximately 70% of the worldwide dromedary population. This can be explained by a ‘restocking from the wild’ scenario, with an initial domestication followed by introgression from individuals from wild, now‐extinct populations (Almathen *et al*. 2016). As suggested by the environmental context in which wild dromedaries would have evolved, i.e. foraging in coastal habitats (Peters 1998), their native distribution and population size were restricted compared with the ancestors of other livestock species. A sudden population decline around 6000–8000 ya (Almathen *et al*. 2016) indicates that, by the time cultural control over the wild one‐humped dromedary was initiated, its populations may already have become increasingly disjointed owing to anthropogenic activities, until they disappeared ca. 2000 ya (Uerpmann & Uerpmann 2002; von den Driesch & Obermaier 2007; Uerpmann & Uerpman 2012; Grigson 2014).

## Dispersal of camels and cross‐species hybridisation

### Migration routes of dromedaries across Africa, Asia and Australia

**Figure 3:**
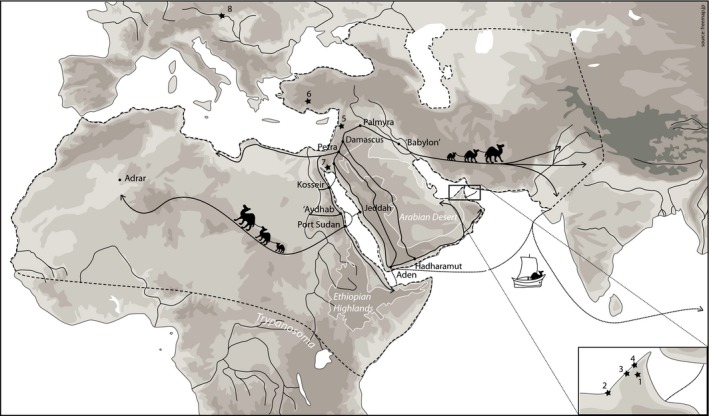
Schematic representation of the historical network of caravan routes (i.e. Incense and Silk routes) according to descriptions from Bulliet (1975) and Heiss (2012). The historical repartition of domestic dromedaries is depicted with dashed lines. Archaeological sites of the ancient specimens used for phylogeographic analyses (Fig. 2) are shown with black stars. Solid lines show the human‐driven camel migration along historic caravan routes: (i) from the Gulf of Aden to the North Arabian Peninsula as part of the Incense Road; (ii) the trans‐Saharan route; and (iii) the Silk Road, which bordered the Mediterranean coast and connected northwestern Africa to the north of the Arabian Peninsula from where caravans departed for southern Asia. The most contemporary migration route started in the 1860s and linked Pakistan to Australia, where several thousand camels were imported. Reprinted from Supplementary Material of Almathen *et al*. (2016).

After their domestication on the Arabian Peninsula, small numbers of dromedaries arrived in Mesopotamia and from there were probably introduced into northeastern Africa via the Sinai, possibly starting in the first millennium BCE. Larger herds in northern Africa appeared only during the fourth to seventh centuries CE (Late Antiquity/Early Middle Ages), where their adoption into local economies may have been slow (Bulliet 1975; Midant‐Reynes & Braunstein‐Silvestre 1977). Another possible route for dromedary introduction into Africa might have involved a transfer from the south of the Arabian Peninsula by boat via the Gulf of Aden to Eastern Africa or further north across the Red Sea to Egypt (Fig. 3). The southern sea route is supported by socio‐ethological observations, as today's Eastern African dromedaries are used largely for milk production rather than for riding and transportation, and this could be rooted in practices associated with the early stages of dromedary husbandry on the southern Arabian Peninsula (Bulliet 1975; Grigson 2012).

Cross‐continental sharing of nuclear genotypes reflects an extensive gene flow between African and Asian dromedaries, notably with a panmictic population on a mitochondrial level (Almathen *et al*. 2016; Lado *et al*. 2018). The traditional usage of dromedaries as pack animals, their exchange and movements along transcontinental caravan routes might account for the observed lack of global population structure. The most contemporary migration route started in the 1860s and linked the Indian Subcontinent to Australia, where several thousand camels were imported until the 1920s for the development of the Australian outback (Faye *et al*. 2004; Rangan & Kull 2010). The historical distribution of dromedaries and their ancient networks of caravan routes (i.e. the Incense and Silk roads) are displayed in Fig. 3.

### Distribution of domestic Bactrian camels in Central Asia

Bactrian camels are distributed mainly in Central Asian countries, including Mongolia, China, Kazakhstan, northeastern Afghanistan, Russia, Crimea and Uzbekistan (Mirzaei 2012; Vyas *et al*. 2015). A few populations can also be found in Northern Pakistan, Iran, Turkey and India (Vyas *et al*. 2015). China harbours the largest number of domestic Bactrian camels, which are located mainly in Inner Mongolia, Xinjiang, Qinghai and Gansu. Phylogeographic analyses of modern Bactrian camels from Mongolia, Russia, Crimea, Kazakhstan, Iran and China revealed shared mitochondrial haplotypes (Silbermayr *et al*. 2010; Ming *et al*. 2017) as well as genome‐wide gene flow across countries (Jirimutu & Ming 2018).

### Hybridisation between dromedaries and domestic Bactrian camels in Western and Central Asia

The distribution areas of dromedaries and Bactrian camels overlap in a few countries in Western and Central Asia, especially in Turkey, Iran, India, Afghanistan, but Kazakhstan is the place where the practice of anthropogenic hybridisation is the most common. In Old World camelids hybridisation between Bactrian camels (*C*. *bactrianus*) and dromedaries (*C. dromedarius*) was associated with the transportation of goods along multiple long‐distance trade routes. This practice intended to produce animals with the robustness of the Bactrian camel, the endurance of the dromedary and an ability to tolerate sharply contrasting climatic conditions. The history of anthropogenic hybridisation is currently being investigated using archaeozoological and palaeogenetic techniques (http://www.hybridcamels.com). Preliminary results revealed hybrids from a Roman archaeological site in Serbia, Viminacium, dated to approximately the late third to fourth centuries CE (Burger *et al*. 2018). A complete camel skeleton from the seventeenth century CE excavated close to the river Danube in Austria attests to the usage of dromedary–Bactrian camel crosses during the second Osmanic–Habsburg war, as troops besieged Vienna (Galik *et al*. 2015).

**Figure 4:**
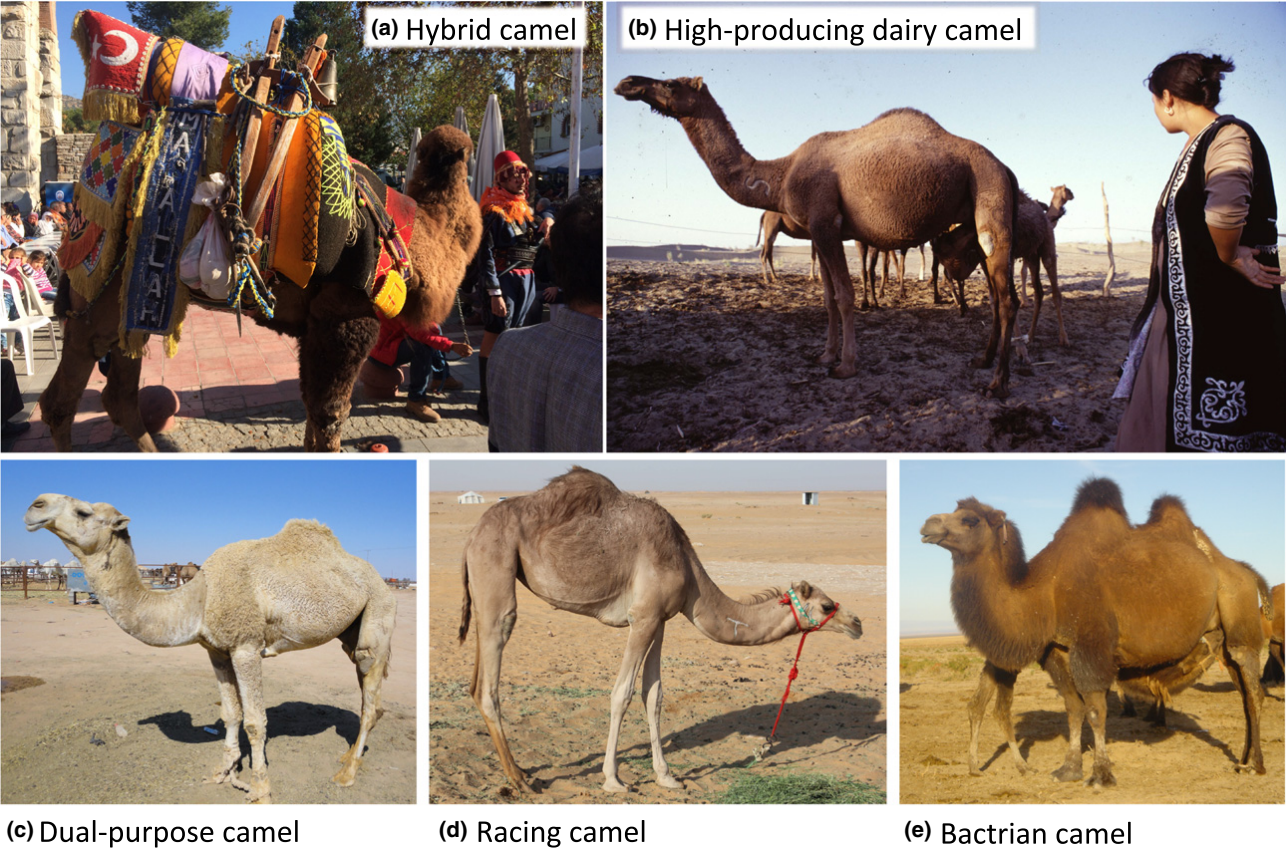
Camel types. (a) Camel hybrid F1 (Bactrian camel × dromedary) used for camel wrestling, a traditional event in Turkey. (b) Arvana dromedary in Turkmenistan. (c) Al‐Homor dromedary in Saudi Arabia. (d) Racing camel Al‐Hurra in Saudi Arabia. (e) Mongolian Bactrian camel with its high‐quality fur. Photo credits: Bernard Faye.

Today, hybridisation facilitates improved milk and wool yield in hybrid Tulu or Nar camels [first generation (F1) hybrids] from Middle Eastern and Central Asian countries. This improvement in physical performance and other (behavioural) traits, termed heterosis or hybrid vigour, arises from allelic interactions between parental genomes, potentially leading to increased growth, productivity and fitness of the fertile F1 hybrids. Hybrids of the second generation (F2), which are crosses between F1 hybrids (Jarbai), in Old World camels are usually not favoured because of their difficult character and weak progeny performance (Faye & Konuspayeva 2012). In western regions of Turkey, a much relished sport is camel wrestling, where prized male Tulus are entred into heavily regulated fights (Çakırlar & Berthon 2014; Manav *et al*. 2018; Fig. 4a).

### Introgression of domestic Bactrian camel in the highly endangered wild two‐humped camels

The wild two‐humped camel population in Mongolia and China appears to be in a steady decline. The latest surveys have revealed a reduction to only 900–1600 animals worldwide (Yadamsuren *et al*. 2019, Lei *et al*. 2012) . The remaining population is surrounded by an estimated number of 10 000 domestic camels, in addition to 50–60 already existing fertile hybrid camels in the circumjacent settlements. In some cases, the hybridisation of domestic females with wild bulls is initiated to enhance the fitness of domestic camels (Yadamsuren *et al*. 2012). The extensive livestock–wildlife interface around the Great Gobi Special Protected Area ‘A’ is of particular concern for the conservation of the Mongolian wild camels. The movement of domesticated animals into the habitat of the wild population leads to the transfer of potential pathogens across this domestic–wildlife interface (Walzer *et al*. 2012). Introgression of domestic Bactrian camel genes into wild camels has been demonstrated in mitochondrial (Silbermayr *et al*. 2010) and nuclear DNA (Silbermayr & Burger 2012) as well as the Y‐chromosome (Felkel *et al*. 2019).

## Purpose, main types and breeds of domestic camels

Old World camels are typically multipurpose animals (Hjört af Ornäs & Hussein 1993). In addition to their utilisation for production as live (milk, wool, manure) or slaughtered (meat, skin, fat) animals, camels are valued for their power in different activities of leisure or work (riding, packing, carting). To facilitate these diverse purposes, humans have selected different types of camels along the domestication process. Roughly eight types have been favoured by camel breeders differentiated by their size (tall/medium/short), global conformation (longilineal/brevilineal) and environment (flat/mountainous areas, sandy/rocky desert) (Blanc & Hennesser 1989). Longilineal animals are used mainly for riding and racing, whereas the brevilineal are employed for packing and other work activities.

Different body conformation measurements have been adopted to describe camel types, however, without any standardised data collection for meta‐analyses. A number of local studies have investigated morphological features and body scores, including heart/barrel girth, height at shoulders/withers and length of body/head/neck/tail with the aim of identifying ecotypes or breeds (Table 1), but with varying success. For example, in Ethiopia, significant morphometric differentiation was achieved for only one (Afar) out of eight pastoralist‐designated populations (Legesse *et al*. 2018). Variation in coat colour has been shown generally not to represent a population distinctive trait (Ishag *et al*. 2011; Abdallah & Faye 2012; Abdussamad *et al*. 2015), despite some dromedary breeds being traditionally named after their predominant coat colour.

**Table 1:** Examples of camel ecotypes/breeds described in literature.

Country	Ecotypes/breeds	Reference
Algeria	Sahraoui, Targui	Oulad Belkhir *et al*. (2013)
Ethiopia	Afar	Legesse *et al*. (2018)
Mauritania	Aftout, Reguebi	Kane (1995)
Morocco	Guerzni, Marmouri, Khouari	Ouassat & Achaabane (1991)
India	Ladakh Bactrian	Makhdoomi *et al*. (2013)
Pakistan	Marecha, Dhatti, Larri, Kohi, Campbelpuri, Sakrai	Shah *et al*. (2014)
Saudi Arabia	Al‐Hurra, Awarik, Awadi, Hadhana, Majaheem, Maghateer, Hamrah, Safrah, Saheli	Abdallah & Faye (2012)
Sudan	Anafi, Kenani, Rashaidi, Bishari, Lahawee	Ishag *et al*. (2011)
Tunisia	Gueoudi, Guiloufi, Merzougui, Ourdaoui,	Chniter *et al*. (2013)
India	Bikaneri, Jaisalmeri, Kachchi, Mewari	Khanna *et al*. (2004) and Metha (2014)
China	Alashan, Sunit, Qinghai, Tarim, Zhungeer, Mulei	Zhao 1998 and Ming *et al*. (2017)
Kazakhstan	Uralobokeliki, Kyzylorda, Ontustik‐Kazakhstan	Terentyev (1975)
Mongolia	Hos Zogdort, Galbiin Gobiin Ulaan, Haniin Hetsiin Huren	Chuluunbat *et al*. (2014)
Russia	Kalmyk	Ming *et al*. (2017)

Indeed, there is lack of uniformity of criteria across countries about definition of breeds, contrary to what is seen in other domestic species. Given their typically multipurpose use and the weak anthropogenic selection pressure, phenotypic diversity is mostly distributed into different eco‐types, rather than breeds, with classification being based mainly on ethnic groups and geographical distribution of the pastoral communities (Legesse *et al*. 2018). Genetic studies, so far, have failed to differentiate distinct breeds and have reported little population structure on a global (Almathen *et al*. 2016, Lado *et al*. 2018) or national scale (Mburu *et al*. 2003; Nolte *et al*. 2005, Schulz *et al*. 2010; Spencer & Woolnough 2010; Chuluunbat *et al*. 2014; Abdussamad *et al*. 2015; Cherifi *et al*. 2017). Genome‐wide analyses on well‐classified populations using standardised phenotyping criteria across countries, however, might identify genetically distinct groups, which could contribute to a novel definition of camel breeds.

### Dairy and dual‐purpose camels

Until recent times, the use of camels for dairy production was a non‐specialised activity and rather a sub‐product as milk was mainly used for the producer's own consumption or sold on the local market. The increased interest in camel milk in a more urbanised world has boosted research activities on dairy camel selection (Faye 2018). Studies have mainly focused on udder morphology (Ayadi *et al*. 2016), management in intensive systems (Nagy & Juhasz 2016) and the assessment of (non‐)genetic factors for milk composition (Nagy *et al*. 2017). Despite the lack of selection pressure for dairy yield in camels, a rough classification into three groups was suggested. (Alhadrami & Faye 2016) The first group is the high‐producing dairy camels with an annual milk production of more than 3000 l are characterised by a large body size (up to 2.40 m at withers) with developed abdomen, large hump, prominent mammary vein and an overall well‐developed udder (Fig. 4b; Table 2). In the second group are medium producers ranging between 1500 and 3000 l are usually dual‐purpose animals (milk and meat or packing/riding) with medium body and hump sizes (Fig. 4c). Such animals are common in the Horn of Africa and the Arabian Peninsula, but also in Sahelian countries (Table 2). Their milk production improvement is less sensitive to feeding supplementation, as these animals have the tendency to accumulate fat in their hump rather than producing more milk. Their coat colour is usually light, contrary to the former group, in which the hair is darker. The third group contains the low producers with less than 1500 l of milk, including the Bactrian camel and dromedaries used for other purposes than milking, thus their udder is not well developed.

**Table 2:** Examples of high‐, medium‐ and low‐producing milk camel ecotypes/breeds.

High‐producing dromedaries		Medium‐producing dromedaries		Low‐producing camels	
Marecha	Pakistan	Hoor	Somalia	Bactrian camel	Central Asia
Al‐Majaheem	Saudi Arabia	Al‐Homor	Saudi Arabia	Maghrebi	North Africa
Sirtawi	Libya	Anafi	Sudan	Manga	Chad, Niger
Arvana	Turkmenistan	Dankali	Ethiopia	Bishari	Sudan
Bikaneri	India	Azbin	Niger	Al‐Shameya	Syria, Iraq
Barrela	Pakistan	Birabish	Mauritania	Anafi	Sudan
Shallageea	Sudan	Waddah	Saudi Arabia		
		Fakhreya	Libya		
		Eyddimo	Somalia		

### Draft power camels

Among the use of camels for their power, a special mention must be given to racing camels. The selection of these animals on their speed, as well as their feeding (highly energetic diet with high‐quality protein), has produced a camel characterised by its light skeleton, fine musculature, narrow abdomen (‘greyhound belly’) and a small hump. The global morphology of racing camel (Fig. 4d) is so specific that in Saudi Arabia they are regarded as a specific breed named al‐Hurra (Faye *et al*. 2011). As for packing animals, usually robust animals are used with short size but a large chest width and a relatively well‐developed hump. Their feet are large and their skin is thicker than in other types. Traditionally, for historic caravans like the ‘Silk Road caravan’, merchants used hybrids between dromedary and Bactrian camels (Faye & Konuspayeva 2012; Fig. 4a).

### Camels for meat production

In some countries, mainly young males up to two years old (named *hachi* in Arabian countries) are slaughtered for meat, whereas in other regions adults are preferred (Faye 2013). These preferences have led to different fattening systems: (i) extensive, pastoral fattening mainly used for adults, e.g. in Somalia and Ethiopia; and (ii) intensive fattening with feedlots for young camels as practised, for example, in Saudi Arabia, United Arab Emirates and Tunisia.

A unique case of the use of an invasive species is represented by the feral dromedary population in Australia. Imported between 1837 and 1907 from Afghanistan and Pakistan (Stevens 1989), they were used for establishing infrastructure in agriculture and mining (McKnight 1969). After mechanisation of agriculture, dromedaries were abandoned into the wild where they increased in numbers to a currently estimated 1 500 000 animals. Today, they are captured for meat production within the emerging Australian camel sector and for export to the Arabian Peninsula (Spencer & Woolnough 2010; Spencer *et al*. 2012).

### Wool‐producing camels

Wool production is mainly found in Bactrian camels, especially in Mongolia and China. The high‐quality wool of these breeds is valorised on the international wool market. In Mongolia, some breeds, e.g. Hos Zogdort from Gobi–Altai province (Chuluunbat *et al*. 2014), have been selected for their wool production (Fig. 4e). Wool colour and yield together with body conformation, carcass traits, work power and milk yield for the four major Bactrian camel types in China identified the Alashan Bactrian camel as the top wool producer, with a maximum of 12 kg in males and about 6 kg in females (Zhao 1998).

## Investigated phenotypes: morphology, production and other traits

The lack of systematic animal identification and recording for production traits has made morphology the primary descriptor so far. The latest initiatives aim at unifying and facilitating easy phenotype collection via smartphone applications (Alhaddad & Alhajeri 2018, 2019). Among the investigated phenotypes, which are of high interest for camel breeders and scientists, are milk, meat and reproduction traits, the gut microbiome and the immune system.

### Dairy production traits and performance

Only a limited number of studies have addressed the characterisation of Old World camelids’ production performances at the population level. Dromedary milk production has been investigated in multiple countries (e.g. Saudi Arabia: Musaad *et al*. 2013; Aziz *et al*. 2016, Tunisia: Jemmali *et al*. 2016), but using different frequencies in milk recording as well as lactation lengths, thus making comparisons not straightforward. Indeed, the lack of standardised recording methods and lactation lengths, together with the heterogeneity of the farming system, especially in extensive management, has hampered a comprehensive analysis of the dromedary milk production potential on a global scale (Faye 2008). Recently, Nagy *et al*. (2017) monitored the changes in milk gross chemical composition of individual dromedaries representing seven different populations over a 5‐year period, showing a strong influence of the respective dromedary types on all parameters. Furthermore, the milking performance of three Saudi dromedary types managed under the same conditions during a 10‐month lactation period showed that camels achieved peak yields at the fourth month of lactation, whereas the total lactation yield and milk composition varied among the three populations (Gaili *et al*. 2000).

Concerning dromedary milk composition, significant differences in freezing point, conductivity, milk yield, fat, lactose, ash, solids non‐fat and protein were documented among four dromedary populations from Sudan (Elobied *et al*. 2015). These findings were consistent with a ‘breed’ effect in milk chemical composition between four dromedary populations from Saudi Arabia (Aljumaah *et al*. 2012) and with a wide range of variation in fatty acids profiles and milk protein sub‐units in dromedaries from Jordan (Ereifej *et al*. 2011). Finally, dromedary udder morphology was investigated in a number of studies, highlighting clear variation in the udder, teat shapes and dimensions, and their relationship with milk yield in lactating animals (Eisa 2006; Ayadi *et al*. 2013, 2016; Atigui *et al*. 2016; Mostafa *et al*. 2017; Musaad *et al*. 2017).

Overall, most of the available literature has focused more on non‐genetic factors (Shuiep *et al*. 2014; Bakheit *et al*. 2015) than on genetic factors of phenotypic variability. In view of the importance of camels for milk production in many regions with increasing desertification, we identify the traits milk yield, milk gross composition and udder morphology as prime targets for future genomic selection using the recently developed genomic tools.

### Milk proteins and related genes

Recently, advanced proteomic techniques have been used to analyse the proteome of dromedary and Bactrian camel milk whey. As in cow milk ca. 80% of the total protein fraction of camel milk is represented by caseins (CN), consisting of *α*_s1_‐, *α*_s2_‐, *β*‐, *γ*‐, and *κ*‐CN (Erhardt *et al*. 2016; Ryskaliyeva *et al*. 2018; Singh *et al*. 2018), as well as a *β*‐CN short isoform (Ryskaliyeva *et al*. 2018). Whereas *β*‐ and *κ*‐CN were monomorphic, three different genetic variants (A, C and D) were identified in the *α*_s1_‐CNs (Erhardt *et al*. 2016). Overall, milk whey proteins were reported to display a wide range of bioactivities including immune modulating, antibacterial and antifungal activities (reviewed in Mati *et al*. 2017). In relation to milk production, oxytocin is a neurohypophysial peptide linked to milk ejection, temperament and reproduction. The novel characterisation and re‐sequencing of the 825 and 811 bp long *OXT* gene in dromedaries and Bactrian camels showed one and two polymorphisms in the intron regions of this gene, respectively. These results provide the basis for future association studies for milk and reproduction traits (Pauciullo *et al*. 2018).

### Meat performance and composition

Meat performances and composition according to camel type were investigated in four Saudi Arabian dromedary populations using carcass traits, and physical, chemical and organoleptic meat traits (Al‐Atiyat *et al*. 2016). A wide range of variation was observed for most of the variables between the four dromedary types, with a clear differentiation of Majaheem from the other three dromedary types (Maghateer, Hamrah and Safrah). The effect of thermo‐alkaline treatment in reducing hepatotoxin contamination in camel meat has been demonstrated (Tan *et al*. 2016). Whereas myostatin gene structure, polymorphism and expression in dromedaries have been characterised (Favia *et al*. 2019), no (genome‐wide) association study has been performed for growth or meat performance.

### Reproduction

Female and male reproductive performances according to camel type were traced in three Indian dromedary populations (Deen 2013). Several parameters, like conception rate, first service conception rate, percentage of infertile females, average number of services required per fertile female, pregnancy rate, sperm morphology and motility, and testosterone profiling, were shown to exhibit inter‐type variability. The latest study on pregnancy and parturition in over 2100 dromedaries from six different breeds/ecotypes showed that the season (month of the year) and the female camel (not the breed) were the most important determinants of variation in gestation length and calf birth weight. Seasonal changes were independent of nutritional factors but associated with climatic conditions, e.g. the photoperiod (Nagy & Juhasz 2019). Assisted reproduction technologies are mainly practiced in racing dromedaries, as newly developed techniques are labour‐ and cost‐intensive. Usually, embryo transfer is favoured over artificial insemination owing to the high viscosity and difficulty of preservation of semen in Old World camels (for a detailed review see Skidmore 2019).

### Gut bacterial communities

Camels are pseudo ruminants as their stomach consists of only three ventricles, partially corresponding to the four‐ventricle system in other ruminants (Wang *et al*. 2000). Recent studies on the camel gastrointestinal tract metagenome detected at least 27 bacterial phyla. Whereas in the forestomach a higher number of bacteria were associated with amino acid metabolism, replication and repair, carbohydrate metabolism was enriched in the large intestine and faeces (Gharechahi & Salekdeh 2018; He *et al*. 2018). A novel thermo‐stable xylanase showing a high activity in a broad pH and temperature range was discovered in the dromedary rumen metagenome, suggesting a potential application in some industrial sectors (e.g. camel faeces paper, biofuel, textile, green plastics industry; Ariaeenejad *et al*. 2019).

### Immune genes

Old (and New) World camelids are considered unique among mammals because of several peculiarities in their adaptive immune response. In addition to conventional antibodies, i.e. IgGs that usually consist of two identical heavy (H) and two light (L) chains, camelids have functional homodimeric IgGs composed of only two identical H‐chains, but missing the L‐chains. The antigen‐binding region of the so‐called Nanobody^®^ is reduced to a single variable domain of the H‐chain (VHH) (Riechmann & Muyldermans 2000). Nanobodies have successfully been applied in research, e.g. for cancer therapy, as they revealed beneficial biophysical and pharmacological properties for *in vivo* applications (Muyldermans *et al*. 2009; for a detailed review see Ali *et al*. 2019).

Somatic hypermutations in T‐cell receptor *δ* and *γ* genes increase the diversity repertoire of T‐cells in dromedaries. They have not been identified in mammalian species so far and could enhance the acquirement of new antigenic specificity (Ciccarese *et al*. 2014). On the contrary, *α* and *β* T‐cells show a reduced repertoire with great sequence identity between orthologous genes in all three Old World camel species (Antonacci *et al*. 2019). This might be due to equally limited requirements of the *αβ* CDR1 and CDR2 domains, which bind to the MHC molecules; these in return show low levels of genetic diversity (Plasil *et al*. 2016, 2019). In Old World camels the MHC is located on the long arm of chromosome 20; its general structure, MHC class II – MHC class III – MHC class I, resembles that of other mammalian species (Plasil *et al*. 2016, 2019).

### Disease and environmental adaptation

Several surveillance studies have been carried out addressing known (Tehseen *et al*. 2015; El Wathig *et al*. 2016) and emerging (Miguel *et al*. 2017; Babelhadj *et al*. 2018) zoonotic diseases, like the Middle Eastern Respiratory Syndrome (reviewed in Mubarak *et al*. 2019). The importance of proteomic studies to understand camel adaptation to desert environment has been highlighted (Warda *et al*. 2014), as well as the cellular and molecular mechanisms in response to heat stress (Hoter *et al*. 2019). To the best of our knowledge, no study has investigated genomic aspects in dromedaries or Bactrian camels related to disease resistance, resilience or environmental adaptation.

## Current molecular resources (reference genomes, other datasets)

### Whole genome resources

Presently there are six whole genomes for Old World camels publicly available at GENOME‐NCBI (https://www.ncbi.nlm.nih.gov/genome), assembled using Illumina short paired‐end and <20 K insert mate‐pair reads on different scaffold levels. The first genome was published for *C. ferus* in 2012 (GCA_000311805.2, Jirimutu *et al*. 2012) followed by two domestic Bactrian camel genomes (GCA_000604445.1, Burger & Palmieri 2014 and GCA_000767855.1, Wu *et al*. 2014). Two of the three available dromedary genomes (GCA_000767585.1, Wu *et al*. 2014 and GCA_000803125.1, Fitak *et al*. 2016a,b) show a high assembly quality (few scaffolds, long N50), whereas the third genome is more fragmented (GCA_001640815.1, Alim *et al*. 2019). Improved dromedary genome assemblies on a chromosome level using a combination of Illumina short reads, PacBio reads, Dovetail Hi‐C/Chicago and 10X Genomics Chromium sequencing, respectively, have been presented (Brooks *et al*. 2019) and are available now (Elbers *et al*. 2019). Further efforts to develop a high‐resolution dromedary genome map involve two radiation hybrid panels at different resolution (5000RAD and 15000RAD; Perelman *et al*. 2018) to be sequenced at low coverage.

### Transcriptomes

At the transcriptomic level, a first screening of *C. dromedarius* ESTs representative of 11 tissues (brain, liver, kidney, heart, muscle, lung, spleen, pancreas, stomach, genitals and colon) produced a set of 23 602 putative gene sequences out of which over 4500 were potentially novel or fast evolving gene sequences (Al‐Swailem *et al*. 2010). A transcriptome shotgun assembly from 10 Indian dromedary tissues is available on NCBI (PRJNA82161) and a catalogue of transcripts resulting from an RNAseq experiment performed in seven tissues (brain, kidney, liver, lungs, muscle, skin and testis) was released in a database (http://14.139.252.118/Dcamel/index.php, Prasad *et al*. 2014). A recent *de novo* dromedary transcriptome assembly has been presented (Holl *et al*. 2018) and will probably be released soon.

### Re‐sequencing projects

Ongoing re‐sequencing projects involve multiple populations of both dromedaries and Bactrian camels with the goals of investigating genome‐wide diversity, population structure, demography, and signals of selection (Fitak *et al*. 2016a,b; Al Abri *et al*. 2017; Jirimutu & Ming 2018), and finally to detect variants for the development of a genotyping platform. To achieve this aim, more than 400 whole genomes of Old World camels representative of the entire distribution range will be sequenced in the first worldwide camel diversity study offered by the 2019 Illumina Agricultural Greater Good Initiative grant (to E. Ciani). This will be a first step towards the development of an Illumina^®^ CamelHD BeadChip. The sequence information will contribute to deepened understanding of the evolutionary processes shaping camelid genomes and to deciphering the molecular basis of the peculiar physiological adaptation as well as economically important traits of camels.

## Current challenges and future perspectives

There is a growing research community active in different aspects of camel physiology and genetics, which is timely in view of the increasing demand for camel products all over the world. New establishments like the International Camel Consortium for Genetic Improvement and Conservation funded under the umbrella of the International Society of Camelid Research and Development, now counting over 80 members from various countries, or EU project like ‘Towards a CAmel tRAnsnational VAlue chaiN (CA.RA.VA.N)’ and ‘CAMELMILK’, as well as a new International Committee for Animal Recording initiative (ICAR), aim to establish the *status quo* of animal identification and performance recording in Old World camels, and to develop guidelines on an international scale.

In the medium term, the availability of a camel SNP genotyping platform may boost national governments’ investments in national breeding programmes based on systematic phenotype and genealogical recording. Such data will form the basis for improved breeding practices and breed management, and for future estimation of genomic breeding values and genomic selection (e.g. Hayes *et al*. 2010) using a training population of a minimum of 1000 phenotyped (e.g. milk yield and cross composition, growth, disease resistance) and genotyped dromedaries. Furthermore, the available genomic resources can be applied to monitor diversity, population structure, inbreeding and admixture in the domestic dromedary and Bactrian camels. In particular, there is a need to genetically monitor the critically endangered wild two‐humped camels in Mongolia and China. Future studies should target the identification of genomic regions important for the adaptation of wild camels to their specific environments and to ensure their conservation as last wild representatives of the Camelus family.

In the long term, the challenge remains to harmonise and standardise the collection of phenomic and genomic data und to utilise them in a way that is beneficial for human and animal needs. This includes not only the improvement of desirable production traits but also the conservation of genomic diversity and of the evolutionarily significant physiological adaptations in camels.
